# Characterization of influenza A(H1N1)pdm09 isolates of Peru using HRM, a post PCR molecular biology method

**DOI:** 10.6026/97320630015640

**Published:** 2019-10-10

**Authors:** Priscila Lope, Huaringa Maribel, Mayta Egma, Bailon Henri, Padilla Carlos

**Affiliations:** 1 Laboratorio de Referencia Nacional de Virus Respiratorio, Centro Nacional de Salud Publica, Instituto Nacional de Salud, Lima, Peru; 2 Laboratorio de Referencia Nacional de Biotecnologia y Biologia Molecular. Centro Nacional de Salud Publica. Instituto Nacional de Salud. Lima. Peru; 3 Laboratorio de virologia. Universidad Nacional Mayor de San Marcos. Lima. Peru

**Keywords:** High Resolution Melting, H1N1 Influenza Virus Subtype A, Genotype

## Abstract

Influenza caused by A(H1N1)pdm09 is a public health issue with severe conditions in vulnerable populations leading to death. Therefore, it is of interest to characterize and monitor influenza A(H1N1)pdm09 genotypes using High Resolution Melting (HRM), a post PCR molecular biology method. We used HRM analysis (using RotorGene Q thermocycler) to characterize A(H1N1)pdm09 genotypes from several places of Peru. RNA was purified from nasal and pharyngeal swab samples referred to LRNVR-INS, synthesized cDNA, and then the hemagglutinin gene and matrix fragment were amplified. Thus, 287 samples positive for influenza A(H1N1)pdm09 were identified across Peru where places like Lima, Piura, and Arequipa documented highest number of cases. The HRM data was analyzed and results showed different profiles which were further grouped into four genotypes for the HA (A, B, C, D) and 3 for the M (a, b, c) genes. We also report ten genotypes (I-X) of virus using combined HA (hemagglutinin) and M gene profiles representing a national geography. The prevalent genotypes are I and II with a frequency of 35.89% (103) and 29.27% (84), respectively linking with severe acute respiratory infection.

## Background

Influenza viruses cause a highly contagious acute respiratory disease. Influenza A virus belonging to the family Orthomyxoviridae, this respiratory pathogen causes annual epidemics and occasional pandemics [1]. Influenza A viruses tend to mutate rapidly and exchange RNA segments between same or different subtypes, generating new virus variants, this rearrangement or exchange of segments between different viruses that co-infect a cell is the main contributor to the increase in diversity and responsible for the most important pandemics [2]. Influenza viruses have a high error rate (1 x 10-3 to 1 x 10-5) during replication due to the low fidelity of RpdR, resulting in mutations in their nucleotide sequence [3,4]. Influenza has de ability to acquire genetic changes that overcome the immunity of previous infections and cause epidemics. The hemagglutinin (HA) and neuraminidase (NA) genes of influenza A viruses usually show high-frequency of variations [5], generating alterations in the antigenic characteristics of surface glycoproteins and susceptibility to antiviral drugs. In Peru, prevalence changes of A(H1N1)pdm09 subtype were observed in the last years. During 2016, 52.2% (795 cases) of influenza confirmed cases were caused by influenza A(H1N1)pdm09subtype. In 2017, few cases of influenza A(H1N1)pdm09 (6 cases, 1.8%) were reported [6]. In contrast in 2018, greater circulation of influenza A(H1N1)pdm09 was reported with a prevalence of 82.7% (956 cases); and for until 2019 June, only 7.8% (24 cases)has reported by LRNVR of Peruvian NIH. Characterization of the influenza virus is an important issue to monitor changes in epidemiological patterns and make decisions in public health. The appearance of new mutations in influenza A(H1N1)pdm09 isolates were reported, that mutations generate antigenic changes in hemagglutinin and drug-resistance alteration in neuraminidase [7-13]. High-resolution melting (HRM) analysis showed capable identify mutations, capable of discriminating gene variations, recognizing viral genotypes [14-18] which is highly consistent with others methods as qPCR and genome sequencing [7-13,19-22]. In the present study, we characterized influenza A(H1N1)pdm09 virus in Peru using HRM analysis.

## Methodology

The nasal and pharyngeal swab samples were referred from several places of Peru to the National Reference Laboratory of Respiratory Virus of Peruvian National Institute of Health during 2015 and 2016, these were diagnosed by real-time RT-PCR as positive for influenza A(H1N1)pdm09 subtype [23]. All samples with Ct less than 25 were chosen to ensure adequate viral load.

## Viral RNA Extraction

For the extraction of viral RNA, the QIAcube automated nucleic acid purifier (QIAGEN) was used with the commercial kit QIAamp® Viral RNA Mini Handbook (QIAGEN) following the manufacturer's instructions.

## High Resolution Melting analysis for determination of influenza A(H1N1)pdm09 virus genotypes

The primers HA-F (5'-CCC AAA GTR AGR GAT CAR GA-3') and HA-R (5'-CCC TTG GGT GTY TGA CAY KT-3') for the hemagglutinin gene were designed using the BatchPrimer3 program v1.0. For the matrix gene, the primers IFU-F (5 '-GCG AGG ACT GCA GCG TAG AC-3') and IFU-R (5'- 'TGA GAC CCA TGC AAC TGG CAA G-3') previously reported [18] were used. cDNA was synthesized by reverse transcription using 0.5 µL of 10 µM reverse primers (HA-R and IFU-R), 1 µL of 10 mM dNTP, 5 µL of nuclease-free water and 5 µL of purified RNA; it was denatured for 5 minutes at 65 °C. 8 µL of the RT reverse transcription reaction (4 µL of 5X RT buffer, 2 µL of 0.1 M DTT, 1.75 µL of nuclease-free water and 0.25 µL of RT SuperScript II RT enzyme) was added and incubated at 42 °C for 50 minutes, then reaction was stopped at 70 °C for 15 minutes; incubations were performed using a conventional thermocycler (Applied Biosystems).

Each gene was amplified using 5 µL of 2X HRM buffer (QIAGEN), 1.4 µL of primers mix (HA-F and HA-R for HA, or IFU-F and IFU-R for M) at 5 µM, 2 µL of cDNA and 1.6 µL of PCR water. The reaction tubes were placed in a RotorGen Q real-time thermocycler (Qiagen) and following thermal conditions was carried out: initial denaturation of 95 °C for 5 minutes, 45 cycles of 95 °C for 10 seconds, 55 °C for 30 seconds and 72 °C for 30 seconds, followed by high resolution melting step from 65 °C to 95 °C at a speed of 0.1 °C/second with acquisition on the green channel. The HRM analysis v 2.3.1 software from RotorGen was used to analyze the HRM profiles. Also, in each experiment A (H1N1)pdm09 influenza culture isolates were used as positive controls, these controls were used routinely for molecular diagnosis by LRNVR-INS. Additional, one Not Template Control was used as contamination control.

## Statistical analysis

Descriptive statistics were applied through the construction of contingency tables and the chi-square test of Pearson of the statistical package Info Stat-Statistical Software Version 5.13.1 was used.

## Results and Discussion:

**Figure 1:**
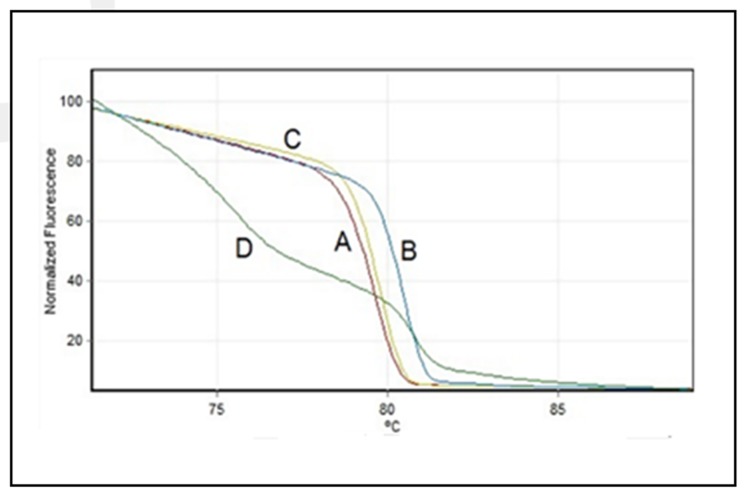
Normalized High-Resolution Melting profiles of Hemagglutinin gene. A, B, C, and D represent the profiles detected in influenza A(H1N1)pdm09 isolates of Peru during the year 2015-2016.

**Figure 2:**
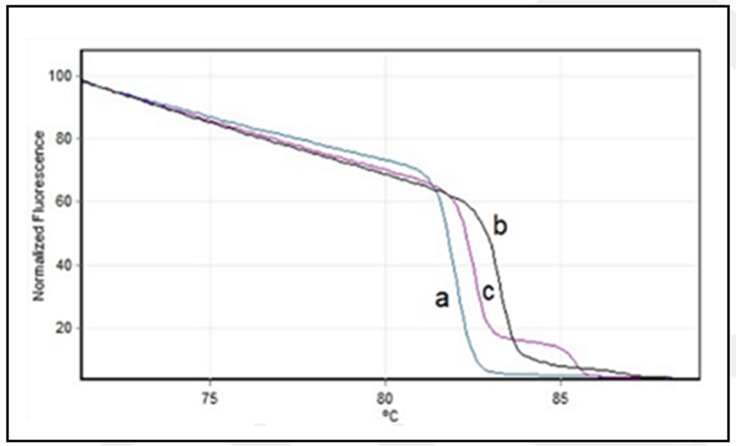
Normalized High-Resolution Melting profiles of Matriz gene. a, b, and c represent the profiles detected in influenza A(H1N1)pdm09 isolates of Peru during the year 2015-2016.

**Figure 3:**
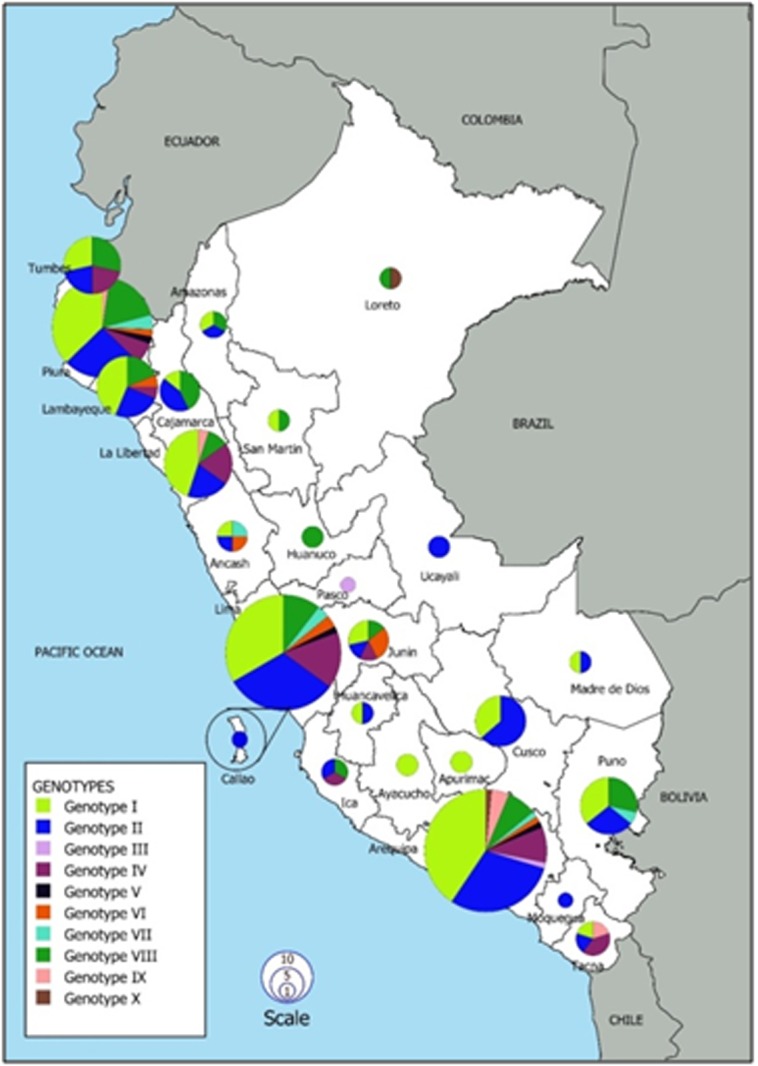
Distribution of genotypes of influenza A(H1N1)pdm09 virus during the years 2015-2016, using the HRM technique using the Matrix and Hemagglutinin genes. Size of circles represents the number of samples according to the scale. The map of Peru is divided by lines that represent the political division of the country. Map derived from QGIS 3.8.2-Zanzibar

788 samples (133 samples of 2015 and 655 of 2016) was reported positive for influenza A(H1N1)pdm09 by the LRNVR-INS using real-time RT-PCR method. From these samples, 287 (31 samples of 2015 and 256 of 2016) were selected because they had an appropriate viral load (Ct <25) for HRM analysis. Samples was classified according to age group: 23% (66) under 5, 13.2% (38) between 5-11, 1.7% (5) between 12-18, 50.5% (145) between 18-59, and 11.5% (33) over 60 years old; according to sex: 48.1% (138) female and 51.9% (149) male; according to clinical classification: 33.8% (97) cases of influenza-like illness (ETI), 54% (155) cases of severe acute respiratory infection (IRAG) and 12.2% (35) cases of severe acute respiratory in health workers (uIRAG). The isolates were grouped into 4 different genotypes using HA gene (A, B, C, and D) (Figure 1) and 3 genotypes using M gene (a, b and c) (Figure 2). This result is consistent with HA mutation rate (0.7-2.6%) which is higher than M mutation rate (0-0.6%) [18].

The HA and M profiles were combined to obtain ten genotypes (I-X) for the analyzed samples. The total samples are grouped with the following frequencies: 35.89% (103), 29.27% (84), 0.7% (2), 10.45% (30), 1.05% (3), 2.79% (8), 2.44% (7), 14.63% (42), 2.09% (6) and 0.70% (2) for genotypes I, II, III, IV, V, VI, VII, VIII, IX and X, respectively. Ten, seven and eight genotypes were detected in Arequipa, Lima and Piura, respectively (Figure 3).Our results reflect the prevalence of cases of influenza A(H1N1)pdm09 in 2015 and 2016 [6] since our sampling was proportional. Therefore, places where the greatest number of A(H1N1)pdm09 genotypes were detected (Piura, Lima, and Arequipa)corresponds to the places with the highest prevalence (Figure 3).In 2016, there was an increase in cases, mainly due to the occurrence of an outbreak in Arequipa [24].

The association between the age groups and the clinical status was evaluated obtaining: cases of children under five years (p = 0.0002), cases between 18 -60 years (p = 0.0001) and cases older than 60 years (p = 0.0013) were associated with IRAG while cases between 5 -11 years are associated with ETI (p = 0.0117). The results obtained indicate that there is no association between age groups and genotypes (p = 0.6807); in the same way, there is no association with sex and genotypes (p = 0.5240); however, we found a significant association between clinical status and genotypes (p = 0.0015). When performing an association analysis with partitions, a significant association was observed between 18-60 age group with genotypes I (p = 0.0001), II (p = 0.0001), IV (p = 0.0026), and VIII (p = 0.0001). The association analysis with partitions between genotypes and clinical classification showed an association between the IRAG status and genotype I (p = 0.0011), genotype II (p = 0.0005), and genotype VIII (p = 0.0055). The HRM analysis has proven to be a flexible, fast, efficient and cost-effective technique to characterize pathogens [14-17]. Also, HRM analysis for characterization of influenza subtypes [18, 25-27] and resistance isolates were reported [10-12,[28-31]. In this study, HRM analysis allowed us to analyze a larger number of samples compared to similar previous studies [32-33], which allowed us to obtain more robust data on the distribution of the most frequent genotypes circulating in Peru. Likewise, our results can be used to monitor changes in the distribution of influenza A(H1N1)pdm09 genotypes over time, like that reported in other studies [7-8,[19-22].

## Conclusion

The High Resolution Melting technique can be used to characterize Influenza A (H1H1)pdm09 isolates which can be applied to molecular epidemiology

## Author contributions:

All authors read and approved the final manuscript
